# Hepatic teratoma and peritoneal gliomatosis: a case report

**DOI:** 10.1186/1757-1626-2-9302

**Published:** 2009-12-10

**Authors:** Christoph Karlo, Sebastian Leschka, Matthias Dettmer, Stefan Breitenstein, Paul Stolzmann

**Affiliations:** 1 Institute of Diagnostic Radiology, University Hospital Zurich, Raemistrasse 100; 8091 Zurich/Switzerland; 2 Institute of Clinical Pathology, University Hospital Zurich, Raemistrasse 100; 8091 Zurich/Switzerland; 3 Department of Visceral Surgery, University Hospital Zurich/Raemistrasse 100; 8091 Zurich/Switzerland

## Abstract

The hepatic teratoma is a very rare entity of which only 25 cases have been published so far. In our case the hepatic teratoma is associated with peritoneal gliomatosis, which is an indicator for an ongoing peritoneal spread of a teratoma. Wall calcifications and the homogeneity as well as the well defined border misled the radiologist to the diagnosis of an echinococcal cyst, which is the most common differential diagnosis, however the hepatic teratoma has to be taking into consideration when dealing with unclear hepatic cysts, although it is very rare.

## Case presentation

### History

In November 2008, a 59-year-old, Swiss Caucasian woman presented to her family physician with mild abdominal pain. No significant events were documented in this previously healthy woman's medical history besides a left sided oophorectomy in 1960 for a teratoma and a hysterectomy in 1982 for unknown indication after three uncomplicated pregnancies. She had no history of trauma and was taking no medication at the time of presentation.

### Imaging findings

**Figure 1:**
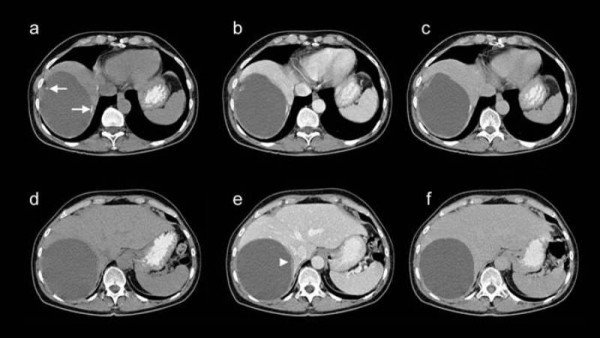
**Transverse computed tomography, non-enhanced (a, d), arterio-venous-enhanced (b, e) and late-enhanced (c, f), images**. Upper row of images demonstrating the calcifications in the cyst's wall (arrows), lower row depicting the enhancement of the cyst's wall (arrowhead).

The general practitioner performed abdominal ultrasound and found an anechoic, well delineated lesion of the right liver, 13 cm (centimeters) in diameter. For further evaluation the patient was referred to our hospital where computed tomography (CT) revealed a homogenous, hypodense, well delineated lesion of the right liver with a diameter of 13 cm and small calcifications in the lesion's wall (Figure 1). The differential diagnoses included an echinococcal cyst, an amebic liver abscess and a cystic necrotic liver metastases of unknown origin. Subsequent laboratory tests for Echinococcosis and Amoebiasis were negative. However, a prophylactic Albendazol therapy was induced. As further growth of this cystic lesion was depicted by a follow-up CT examination three months later (i.e. progression in diameter from 13 cm to 15 cm), a multidisciplinary team meeting decided that a right sided hemihepatectomy needed to be performed and the patient agreed.

### Surgery

**Figure 2:**
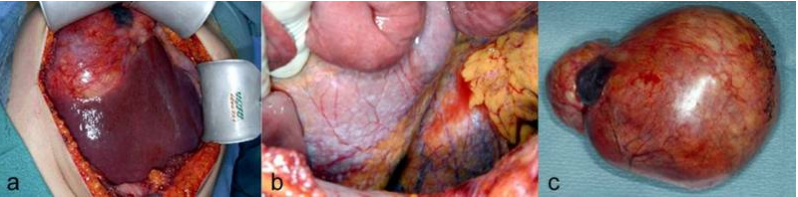
**Intraoperative photograph of the hepatic cyst before resection (a)**. Note the close contact to the liver without macroscopic signs of infiltration. The peritoneum showed multiple white spots (b), later diagnosed as gliomatosis peritonei. Note the encapsulated character of the resected cyst (c).

Intraoperative findings included a large cystic lesion adjacent to the liver without hepatic infiltration as well as multifocal peritoneal thickening combined with white superficial spots on the peritoneum (Figure 2). The cyst was enucleated and multiple biopsies were taken from the peritoneum to evaluate for peritoneal carcinosis or tuberculosis, but neither was confirmed by intraoperative histopathology examinations.

### Histopathology

**Figure 3:**
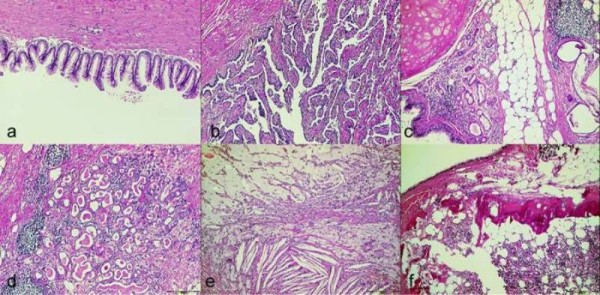
**Different tissue types of the teratoma, which included cells of a mucinous cystadenoma (a), a uterine tube (b), respiratory epithelium, cartilage, fat and glands (c), thyroid tissue (d), cholesterol crystals, cysts with mesothelial covered walls and foam cells (e) as well as bone and bone marrow (f)**.

A large, cystic, well encapsulated, firm-elastic lesion with a diameter of 16 centimeter was macroscopically described by the pathologist and smooth muscle, adipose tissue, hyaline cartilage, thyroid tissue, respiratory epithelium, calcifications and cholesterol crystals were found microscopically (Figure 3). The final diagnosis was a mature, cystic teratoma combined with peritoneal gliomatosis.

## Discussion

Cystic liver lesions are frequent findings in diagnostic imaging and can be of either neoplastic or non-neoplastic origin. We have reported the case of an unusual appearance of a hepatic teratoma in an adult woman combined with peritoneal gliomatosis, which is a very rare, atypical finding. Up to our knowledge of current literature, a total of twenty-five cases of hepatic teratomas have been published so far [1], however radiological imaging studies have not been performed yet.

In our case, the well encapsulated, homogenous, cystic lesion with wall calcifications misled the radiologist initially to the false diagnosis of an echinococcal cyst. Additionally, the intraoperative finding of peritoneal gliomatosis could not be detected on the CT images. Gliomatosis peritonei is thought to be genetically unrelated to the associated teratoma and might be derived from non-teratomatous cells, such as through metaplasia of submesothelial cells [2]. However, the presence of gliomatosis peritonei supports the theory of a peritoneal spread from a previous ovarian teratoma during childhood [3,4]. In our case, the patient underwent oophorectomy at the age of twelve for a left-sided teratoma, therefore the association between teratomas and gliomatosis peritonei can be supported in our case as well.

The key differential diagnoses include the amebic abscess and echinococcal cysts as well as a necrotic metastases, however, in our case the patient had not visited any countries with endemic occurrence of Amoebiasis or Echinococcosis and further on the results of the laboratory examinations were negative for these parasitic disease patterns. A necrotic metastases was ruled out by histopathology.

## Conclusion

We conclude from this reported case that the finding of an unclear, cystic liver lesion and multifocal peritoneal thickening can, although very rarely, represent a hepatic teratoma with associated gliomatosis peritonei and has therefore be taken into consideration as a possible differential diagnosis especially in patients after oophorectomy for a teratoma.

## Consent

Institutional review board approval was granted based on a general waiver, taking into account that patients have the possibility to choose if their data can be used for retrospective investigations. Written informed consent was obtained from the patient for publication of this case report and accompanying images. A copy of the written consent is available for review by the Editor-in-Chief of this journal.

## Competing interests

The authors declare that they have no competing interests.

## Authors' contributions

CK: Image reconstruction, Manuscript writing, SL: Manuscript Editing, Idea, MD: Histology workup, SB: Manuscript Editing, Surgical feedback, PS: Manuscript Editing. All authors have read and approved the final manuscript.
